# Prevalence and Clinical Correlates of Cerebrovascular Alterations in Fabry Disease: A Cross-Sectional Study

**DOI:** 10.3390/brainsci15020166

**Published:** 2025-02-07

**Authors:** Daniele Di Natale, Salvatore Rossi, Gianmarco Dalla Zanna, Antonio Funcis, Tommaso Filippo Nicoletti, Ludovico Luca Sicignano, Elena Verrecchia, Angela Romano, Maria Gabriella Vita, Naike Caraglia, Francesca Graziani, Federica Re, Gisella Guerrera, Luca Battistini, Gabriella Silvestri

**Affiliations:** 1 Department of Neurosciences, Università Cattolica del Sacro Cuore, 00168 Rome, Italy; danieledinatale3@gmail.com (D.D.N.); salvatore.rossi@unicatt.it (S.R.); gdallazanna.gdz@gmail.com (G.D.Z.); antoniofuncis@gmail.com (A.F.); tommasof.nicoletti@gmail.com (T.F.N.); 2 Department of Aging, Orthopaedic and Rheumatological Sciences, Fondazione Policlinico Universitario “A. Gemelli” IRCCS, 00168 Rome, Italy; ludovicoluca.sicignano@policlinicogemelli.it (L.L.S.); elena.verrecchia@policlinicogemelli.it (E.V.); 3 UOC Neurologia Dipartimento Neuroscienze, Organi Di Senso E Torace, Fondazione Policlinico Universitario “A. Gemelli” IRCCS, 00168 Rome, Italy; angela.romano12@gmail.com (A.R.); mariagabriella.vita@policlinicogemelli.it (M.G.V.); naike.caraglia@policlinicogemelli.it (N.C.); 4 Department of Cardiovascular Medicine, Fondazione Policlinico Universitario “A. Gemelli” IRCCS, 00168 Rome, Italy; francesca.graziani@policlinicogemelli.it; 5 Cardiomyopathies Unit, Cardiology Division, St. Camillo Hospital, 00152 Rome, Italy; re.federica77@gmail.com; 6 Neuroimmunology Unit, Istituto di Ricovero e Cura a Carattere Scientifico (IRCCS) Santa Lucia Foundation, 00179 Rome, Italy; g.guerrera@hsantalucia.it (G.G.); l.battistini@hsantalucia.it (L.B.)

**Keywords:** Fabry disease, FD, brain MRI, CNS, central nervous system, stroke, NfL, neurofilament light chain

## Abstract

**Background/Objectives:** Fabry disease (FD) is an inborn error of the glycosphingolipid metabolism with variable kidney, heart, and central nervous system (CNS) involvement. CNS-related FD manifestations include early ischemic stroke and white matter lesions (WMLs) related to cerebral small-vessel disease (CSVD), possibly resulting in cognitive impairment. We studied 40 adult FD patients (17 male) to assess: (i) prevalence of cerebrovascular and cognitive manifestations in FD and their correlation with heart and renal involvement; and (ii) the potential value of serum neurofilament light chain (NfL) levels as an indicator of WMLs in FD. **Methods:** Patients underwent detailed diagnostic assessment related to FD, also including Mainz Severity Score Index (MSSI), neuropsychological tests, brain MRI to assess WMLs by the modified Fazekas score (mFS), and NfL determination by single-molecule array (SiMoA) (*n* = 22 FD patients vs. 15 healthy controls). **Results:** Overall, 4 FD patients had a history of ischemic stroke and 13/32 patients (40.6%) had an mFS ≥ 1. Almost two-thirds of FD patients (27/39, 69.2%) showed impairment on at least one cognitive test. On univariate analysis, only a reduction in estimated glomerular filtration rate was associated with an increased likelihood of having WMLs on brain MRI. Serum NfL levels were higher in FD patients vs. controls, with a trend toward significance (*p* = 0.08). **Conclusions:** Mild-to-moderate CSVD is a characteristic brain “signature” in FD patients. Both cardiac and renal involvement correlate with WML load, but only renal involvement appears to be predictive of CNS damage. Brain microvascular damage is associated with mild cognitive impairment in FD, and serum NfL might represent a potential biomarker of CSVD in FD.

## 1. Introduction

Anderson–Fabry disease (or Fabry disease, FD) is an X-linked inborn error of the glycosphingolipid metabolism caused by mutations in the *GLA* gene, encoding for the lysosomal enzyme alpha-galactosidase A (alpha-Gal A) [1]. The resulting alpha-Gal A deficiency causes lysosomal accumulation of globotriaosylceramide (Gb3) in a wide variety of cells in various tissues, among which progressive involvement of the kidney, the heart, and the central nervous system (CNS) mainly contribute to the global disease burden [2]. FD can occur as an early-onset classical form, usually characterized by the presence of multiorgan involvement, and an atypical late-onset form, where affected individuals manifest with predominant renal or cardiac involvement [1]. In FD patients, the severity of symptoms may vary based on the specific *GLA* pathogenic variant, age, and sex. Generally, pathogenic variants resulting in little to no alpha-Gal A activity cause classical FD, while those allowing residual alpha-Gal A activity cause the atypical phenotype [3]. As with other X-linked diseases, hemizygous males are more severely affected than heterozygous females [4].

Around 80% of FD males manifest the classical phenotype, with neurological, dermatological, kidney, and cardiac manifestations occurring during their second, third, and fifth decades of life, respectively, while atypical FD males present later in life, usually diagnosed during evaluations for cardiomegaly, proteinuria, or stroke [4,5,6]. As the disease pathology progresses, irreversible organ damage eventually occurs, and patients are at risk of life-threatening complications related to kidney, heart, and/or brain involvement [4]. Indeed, many FD patients need renal transplantation, and overall they have reduced life expectancy, mainly due to cardiovascular disease [4,6].

Deriving from accumulating Gb3 in lysosomes, its deacylated form, also known as lyso-Gb3 [7], currently represents the most reliable disease biomarker for FD in affected males, being increased in plasma and urine, while it is not sensitive enough in female FD carriers, both presymptomatic and manifesting with late-onset FD forms [8]. Of note, plasma lyso-Gb3 levels are useful to monitor response to the available disease-modifying treatments [8], and correlate with overall disease severity [9,10], Fazekas score [11] and white matter mean diffusivity measured through diffusion tensor imaging (DTI) [12].

In FD, CNS involvement mainly consists of cerebrovascular manifestations, including early stroke or transient ischemic attack (TIA), and white matter lesions (WMLs) related to cerebral small-vessel disease (CSVD) [13]. A 12-fold higher prevalence of juvenile stroke is observed in FD males (aged 25–44 years) and a 10-fold higher prevalence in FD females compared to the general population [13]: data from the Fabry Registry report that 6.9% of males and 4.3% of females with FD had strokes, mainly ischemic in their etiology [13,14].

Brain MRI shows WMLs in about 46–80% of FD patients [15,16], predominantly located in the subcortical, deep, and periventricular white matter, and usually symmetrically. Cerebral microangiopathy due to Gb3-related arteriolar damage underlies WMLs in FD brains [16]. Most studies agree that accumulation of glycosphingolipids in the vascular endothelium and smooth muscle cells causes various mechanism disturbances in vasoreactivity and autoregulation [14,17]. Finally, cardiac arrythmias, hypertrophic cardiomyopathy, and myocardial infarction may lead to cardioembolic stroke in FD patients, and hypertension due to renal failure might further contribute to increase their stroke risk [18].

WMLs secondary to CSVD can also produce cognitive dysfunction: about 30–41% of FD patients present defects in executive functions, information processing speed, and attention on neuropsychological tests [19,20,21,22]. Psychiatric symptoms, including anxiety and depression, have also been reported in about 50% of FD patients [23,24]. Intraneuronal Gb3 accumulation, which has been described in FD brain tissues [25], could contribute to cognitive decline through direct slowly progressive neuronal cell loss or indirect damage due to astrocyte involvement. Few follow-up studies in FD cohorts have documented a relative stability of cognitive symptoms over time [26].

Given the pleiotropic nature of cerebrovascular brain damage in FD, assessment of determining and potential predictive factors of stroke, WML burden, and cognitive deficit and their mutual relationship in FD is a challenging research topic. Studies conducted so far obtained conflicting results, either due to the small cohort of study or the differences in the diagnostic protocols applied (i.e., brain MRI methodology, neurocognitive test battery).

Therefore, we designed a cross-sectional study on a cohort of 40 adult FD patients being followed up at our institution, aiming to assess: (i) prevalence and clinical correlates of cerebrovascular and cognitive manifestations in FD, taking into account the role that FD-related cardiac or renal manifestations may have in CNS involvement; and (ii) the potential value of known disease biomarkers, i.e., residual alpha-Gal A activity, plasma lyso-Gb3 and serum neurofilament light chain (NfL) levels as indicators of WML load in FD.

## 2. Materials and Methods

### 2.1. Study Design and Clinical Evaluation

From January to December 2023, we consecutively enrolled a cohort of 40 FD patients (17 M, 42.5%) in collaboration with Dr. E. Verrecchia and Dr. L. Sicignano, referring clinicians at the Tertiary Center for Fabry Disease diagnosis (Rare Diseases and Periodic Fever Research Center, Department of Internal Medicine, Fondazione Policlinico Universitario A. Gemelli IRCCS). Inclusion criteria were: age ≥ 18 years, willingness to participate in the study, molecular diagnosis of FD, and availability of diagnostic 1.5 T brain MRI images and of cognitive tests performed within six months from the acquisition of brain MRI scans. The only exclusion criteria consisted in the concurrence of additional CNS damage not directly related to FD (i.e., trauma, brain tumors, others). All patients gave their informed consent to participate to this cross-sectional study. For the diagnosis of FD, according to expert recommendations, we used a combination of phenotypic features, enzymatic assay (male patients), and molecular analysis to reveal pathogenic variants in GLA (all patients) [27].

Clinical disease severity was assessed by the Mainz Severity Score Index (MSSI) at the time of study enrollment. The MSSI scoring system is a validated measure of disease burden specific to FD [28]. It is composed of four sections that cover the general, neurological, cardiovascular, and renal signs and symptoms of FD. The global MSSI score index is given by the sum of the score of each subscore, for a maximum of 76/76.

### 2.2. CNS Involvement

To characterize prevalence and severity of CNS involvement in our FD cohort, we collected the following data.

- History of cerebral events (previous stroke or TIA).
- WMLs, quantified using the modified Fazekas score (mFS) [29] performed on the FLAIR axial sequences of conventional 1.5-tesla brain MRI in 32/40 patients suitable for the examination (not carriers of pacemaker (PMK) or implantable cardioverter device (ICD) as part of their routine follow-up assessment. WMLs were rated on axial FLAIR, ranging from 0 (no WMLs) to 3 (confluent WMLs).
- Cognitive assessment: 39/40 FD patients underwent neuropsychological assessment. The cognitive battery included the following tests with published standardized normative data from large control samples: the Mini-Mental State Examination (MMSE) [30], Rey’s auditory verbal learning task (RAVLT), including subtests of immediate and delayed recall and forced-choice recognition [31], the Rey–Osterrieth figure copy and delayed recall [32], an abstract reasoning test (Raven’s progressive matrices) [31], the Stroop test—short version [31], a demanding visual attention task (multiple features target cancellation) [33], phonological and semantic verbal fluency [31,34], and digit and spatial span forward and backward [35]. Individual test scores were considered either normal or altered based on the normative values. For statistical correlations, only values adjusted for age and education were used where applicable.
- Serum NfL levels were assessed in 22/40 FD patients and in a control group of 15 healthy age- and sex-matched subjects by single-molecule array (SiMoA, Quanterix, Billerica, MA, USA) as previously described [36]. Frozen serum samples were carried in dry ice to the Neuroimmunology Lab, Santa Lucia Foundation IRCCS, Rome (IT) for quantitative determination of NfL.

### 2.3. Other Systems Possibly Influencing CNS Impairment

To investigate potential predictors of WML load in FD, we collected the following data for the statistical analysis for each patient.

- Age at evaluation (AE), sex at birth, years of education.
- Type of *GLA* variant.
- Phenotype (classic or late onset).
- Age at onset (AAO) of FD symptoms and years of disease duration (DD).
- Type and years of treatment (enzymatic replacement therapy (ERT) or chaperone).
- Plasma lyso-Gb3 levels 6 months before and 1 year after starting treatment. Plasma lyso-Gb3 levels were measured by tandem mass spectrometry [37] at an external laboratory (Centogene GmbH, Rostock, Germany).
- Residual enzymatic alpha-Gal A activity (measured on leukocytes and expressed as percentage of the normal mean).
- Renal function parameters (estimated glomerular filtration rate (eGFR, expressed in mL/min), creatinine (mg/dL), blood urea nitrogen (BUN, mg/dL), cystatin C (mg/dL), 24 h albuminuria (mg) and 24 h proteinuria (mg)), presence of renal dysfunction (eGFR < 90 mL/min or 24 h proteinuria > 300 mg/die or spot proteinuria >30 mg/dL), severe renal dysfunction (eGFR < 30 mL/min/1.73 m^2^, dialysis or renal transplantation). eGFR was calculated using the Chronic Kidney Disease Epidemiology Collaboration equation [38].
- Common vascular risk factors and comorbidities (hypertension and use of antihypertensive medications, dyslipidemia and use of statins, type 2 diabetes mellitus, smoking).
- Presence of cardiac hypertrophy and major cardiac events (atrial fibrillation or any major rhythm disturbance, congestive heart failure, implantation of an ICD or PMK, myocardial infarction, coronary artery bypass graft (CABG) surgery, or a percutaneous transluminal angioplasty). Echocardiographic parameters included: interventricular septum (IVS) and left ventricle (LV) posterior wall thickness, end-systolic and end-diastolic LV diameter, 2D guided B-mode calculated left ventricle mass index (LVMi), and relative wall thickness (RWT). Left ventricular hypertrophy was defined as a left ventricular mass index (LVMi) higher than the upper limit of normal (men ≥ 103 g/m^2^, women ≥ 89 g/m^2^) [39].
- Presence of subjective depressive symptoms and use of antidepressants.
- Diagnosis of migraine/tension headache.
- Severity of pain assessed by VAS (visual analogue scale).
- Use of medications for neuropathic pain, presence of acroparesthesias and/or dyshidrosis.
- Presence of angiokeratomas and corneal abnormalities, tinnitus, and/or dizziness.
- Presence of polyneuropathy detected by nerve conduction studies (NCSs) on lower limbs. NCSs were performed using Natus Keypoint EMG equipment (Middleton, WI, USA). Antidromic sural SNAP, tibial CMAP and F wave latency were measured and compared to our reference values [40].

### 2.4. Statistical Analysis

The sample was characterized on clinical and demographic features using descriptive techniques. Quantitative variables are described using mean and standard deviation (SD), median, minimum (min.), and maximum (max.). Qualitative variables are summarized with absolute and percentage frequency tables. Normality of continuous variables was checked using the Kolmogorov–Smirnov test. N-value was specified for each variable. Patients with some missing values were included in the study and maintained as missing. The Mann–Whitney U test was used to evaluate the presence of differences for continuous variables between FD patient subgroups (i.e., males vs. females). This test was also used to evaluate differences in serum NfL levels between FD patients (*n* = 22) and age-matched healthy controls (*n* = 15). The χ^2^ test or Fisher’s 2-tailed exact test were used to compare categorical variables between the subgroups (males vs. females, classical vs. atypical late-onset cases). Both mFS scores and NfL levels were correlated by Pearson’s correlation test (or Spearman’s correlation test if required) with demographic variables (AE), FD-related features (AAO, DD, years of treatment, enzymatic activity percentage, lyso-Gb3 levels), cardiac (IV septum thickness, posterior wall thickness, LV end-diastolic diameter, LV end-systolic diameter, RWT) and renal indices (serum creatinine, cystatin C and BUN levels, eGFR, 24 h proteinuria and 24 h albuminuria level), neuropsychological test scores.

A binomial logistic regression was performed to evaluate the effects of age, gender, eGFR, and known risk factors for cardiovascular disease (smoking, diabetes mellitus, systemic hypertension, hypertrophic cardiopathy) on the likelihood of having leukoencephalopathy, defined as mFS ≥ 1. A stepwise method with a forward selection approach was used to perform variable selection. Significance was set at *p* < 0.05. Statistical analysis was performed using SPSS (Statistical Package for Social Science, IBM SPSS Statistics, version 29.0. IBM Corp.: Armonk, NY, USA).

## 3. Results

### 3.1. Demographic and Clinical Characteristics of FD Patients

The study cohort included 40 FD patients (mean age 45.38 ± 16.93 years), with a non-significant female prevalence (23/40, 57.5%) (Table 1). Pathogenic GLA variants found in our FD cohort are summarized in Supplementary Table S1.

Out of the total, 24 patients (60.0%) exhibited a classical phenotype and 16 (40.0%) a late-onset atypical phenotype. At the time of assessment, 32/40 (80.0%) patients were under specific FD treatment (25 ERT, 6 chaperone). Out of the eight untreated patients (two males and six females), one male had refused the therapy, one had not yet started it even though it was recommended, and the six women did not meet the followed criteria [39].

Mean age at onset of symptoms was 26.90 ± 17.53 years and DD was 19.41 ± 17.30 years, without significant differences regarding gender (Supplementary Table S2). Mean age at diagnosis was 35.35 ± 17.83 years. Mean duration of therapy was 8.50 ± 5.53 years.

Mean alpha-Gal A activity prior to the initiation of treatment (available for 39/40 patients) was 28.86 ± 21.68% of normal values. FD males showed significantly lower activity values compared to female (Supplementary Table S2).

Lyso-Gb3 level before starting therapy (available for 34/40 patients) was above the reference range (0–1.8 ng/mL) in 31/34 patients (91.209%), with a mean value of 11.27 ± 14.58 ng/mL. Lyso-Gb3 levels were significantly higher in males vs. females (Supplementary Table S2).

Mean lyso-Gb3 level after starting therapy (available for 34/40 patients) decreased to 8.90 ± 12.08 ng/mL, still maintaining significant differences between males and females.

Out of 40, 9 FD patients (22.5%) had experienced major cardiac events (i.e., atrial fibrillation or any rhythm disturbances, congestive heart failure, implantation of an ICD or pacemaker, myocardial infarction, CABG, or a percutaneous transluminal angioplasty), and 2/40 (5.0%) had atrial fibrillation and were on anticoagulant therapy (Table 1).

Of 40, 8 (20.0%) had a PMK/ICD implanted and 3/40 (7.5%) a loop recorder.

Of 40, 9 (22.5%) were on antithrombotic therapy (low-dose aspirin).

Of 40, 22 (55.0%) had systemic arterial hypertension and were on antihypertensive therapy, 2 (5.0%) had diabetes mellitus, 12 (30.0%) had dyslipidemia, 11 (27.5%) were on statins, and 16 (40.0%) were smokers.

For all 40 patients, transthoracic echocardiography was performed within 6 months before or after study enrollment.

Mean IVS thickness was 13.29 ± 5.57 mm and mean posterior wall thickness was 11.52 ± 3.55 mm. Mean LV end-diastolic diameter was 44.5 ± 8.85 mm, and mean LV end-systolic diameter was 27.06 ± 4.6 mm. Mean RWT was 0.45 ± 0.16 cm.

The mean value of LVMi was 123.50 ± 60.86 g/m^2^. Out of 40 patients, 18 (45%) had an LVMi value higher than the upper limit of normal (men ≥ 103 g/m^2^; women ≥ 89 g/m^2^), consistent with cardiac hypertrophy [39].

Mean eGFR was 89.12 mL/min/1.73 m^2^ ± 33.83. Mean creatinine, BUN, and cystatin C levels were 1.01 mg/dL ± 0.47, 19.45 mg/dL ± 10.91, and 0.99 mg/dL ± 0.52, respectively (Table 1).

In sum, 20/40 had renal insufficiency (eGFR < 90 mL/min/1.73 m^2^) and 3/40 (7.5%) had experienced major renal events (i.e., eGFR < 30 mL/min/1.73 m^2^, dialysis or renal transplantation).

The mean level of 24 h proteinuria (*n* = 26) was 63.48 mg/dL ± 126.33. The mean level of 24 h albuminuria (*n* = 37) was 130.64 mg/dL ± 290.63.

Mean global MSSI score was 17.80 ± 14.30 (mean general score 4.08 ± 3.17, mean neurological score 4.38 ± 3.49, mean cardiovascular score 5.48 ± 5.79, mean renal score 3.55 ± 4.54) (Table 1), with a significantly higher global score in males vs. females. Males showed significant higher cardiac (*p* < 0.001) and renal subscore values (*p* = 0.025) (Supplementary Table S2).

Accordingly, ultrasound parameters indicative of hypertrophic cardiomyopathy showed higher pathological values in males vs. females (Supplementary Table S2). PMK/ICD implantation was also more frequent in males (*p* = 0.006) (Supplementary Table S2).

For renal parameters, eGFR, creatinine, and cystatin C values showed significant pathological values in FD males, while 24 h proteinuria and albuminuria and occurrence of major renal events were comparable (Supplementary Table S2).

Among general cardiovascular risk factors, hypertension was more frequent in FD males (*p* = 0.019) (Supplementary Table S2).

Prevalence of other clinical manifestations is also detailed in Table 1. When comparing male vs. female FD forms, none of these manifestations showed significant differences for presence or severity (Supplementary Table S2).

### 3.2. Prevalence and Correlates of Cerebrovascular Manifestation, WMLs, and Cognitive Impairment

A total of 4 out of 40 patients (10.0%) had had an ischemic stroke (2 large-vessel, 2 lacunar) (Table 1). Of these 4 patients, only one with a large-vessel ischemic stroke had had also multiple TIAs. Incidence of stroke was not different between sexes (Supplementary Table S2), but seemed more prevalent in late-onset patients, as all 4 strokes happened among the 16 late-onset patients (25.0%) vs. none among the 24 classical patients (0%), *p* = 0.020.

Regarding WMLs, 13/32 (40.6%) had an mFS ≥ 1 and 6/32 (18.8%) ≥ 2. mFS was 1 in seven patients (21.9%), 2 in three patients (9.4%), and 3 in three patients (9.4%). Overall, mean mFS was 0.69 ± 1.00.

No differences in severity of the mFS or MSSI neurological subscore were found concerning sex (Supplementary Table S2). Moreover, mFS did not significantly differ between treated (either with ERT or chaperone *n* = 25, mFS = 0.80 ± 1.08) and untreated FD patients (*n* = 7, mFS = 0.29 ± 0.49, *p* = 0.395), which also showed comparable mean AE (*n* = 32 treated patients AE = 47.72 ± 16.40 years vs. *n* = 8 untreated patients, AE = 43.97 ± 23.06 years, *p* = 0.475).

Overall, 39 out of 40 patients underwent neuropsychological tests battery (1 patient refused to undergo this examination). Descriptive statistics of all neuropsychological tests scores are illustrated in Table 2.

**Figure 1:**
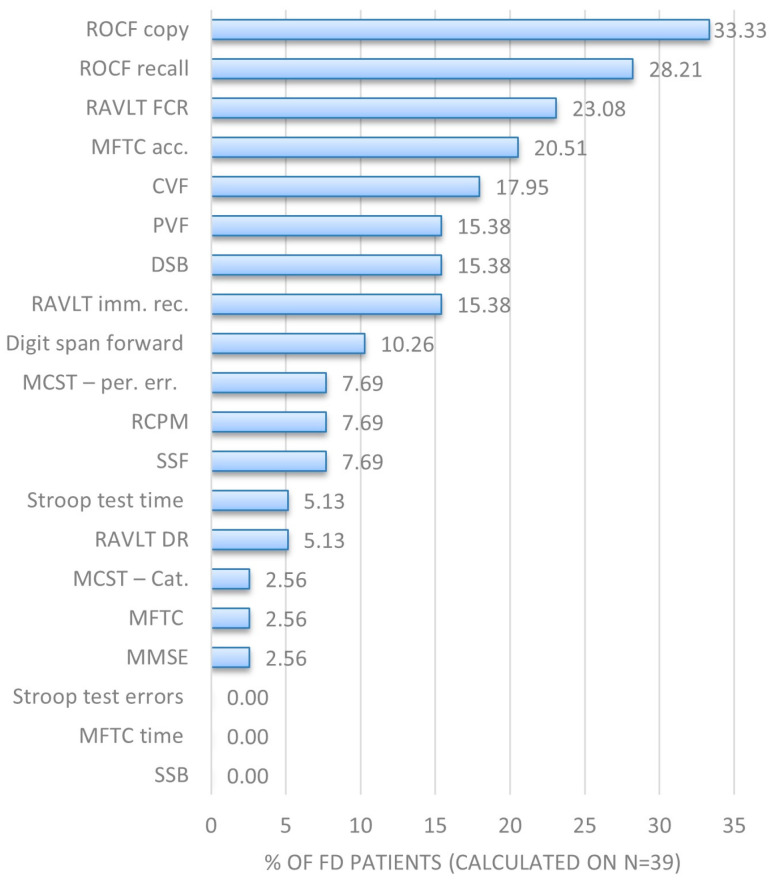
Histogram showing, for each neuropsychological test, percentage of FD patients with abnormal values. Abbreviations: acc., accuracy; cat., category; CVF, categorial verbal fluency; DR, delayed recall; DSB, digit span backward; FCR, forced-choice recognition; imm. rec., immediate recall; per. err., perseverative errors; PVF, phonological verbal fluency; RCPM, Raven’s colored progressive matrices; ROCF, Rey–Osterrieth complex figure; SSB, spatial span backward; SSF, spatial span forward.

Overall, 27/39 patients (69.2%) showed impairment on at least one test. Tests most frequently altered (considering adjusted score, where applicable) were the copy and delayed recall of Rey’s complex figure (13/39, 33.3% and 11/39, 28.2%, respectively), multiple feature target cancellation (MFTC) test accuracy (8/39, 20.5%), semantic and phonological verbal fluency tests (7/39, 18.0%, and 6/39, 15.4% respectively), Rey auditory verbal learning test (RAVLT) forced-choice recognition (9/39, 23.1%) and immediate recall (6/39, 15.4%), and the forward and backward digit-span tests (4/39, 10.3%; and 6/39, 15.4%, respectively). The percentages of impaired performances for single tests are illustrated in Figure 1.

No differences were found between FD males and females regarding the cognitive profiles found.

### 3.3. Serum NfL Levels

**Figure 2:**
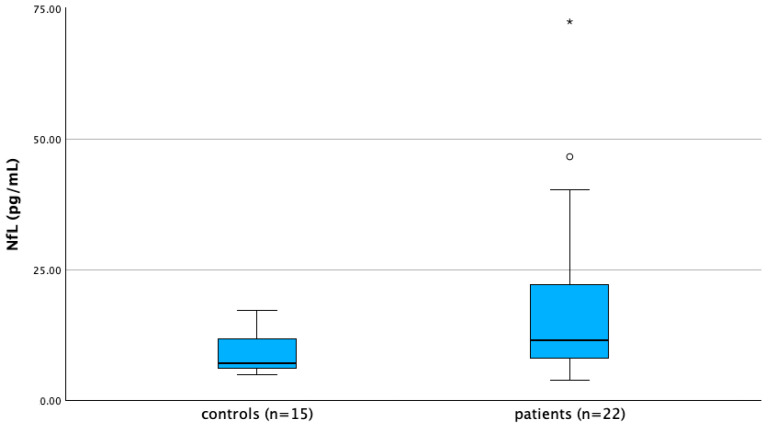
Box plot showing serum neurofilament light chain (NfL) levels in patients vs controls. Please note that the extreme outlier described in the text with NfL levels >600 pg/mL is not included in the graph to better visualize data on the y axis reference scale. * represent an outlier.

Serum NfL levels were higher in FD patients (*n* = 22, mean 45.59 ± 129.36, median 11.50, IQR 17.65 pg/mL) compared to controls (*n* = 15, mean 9.32 ± 4.33, median 7.12, IQR 6.65 pg/mL), with a trend toward significance (*p* = 0.08) (Figure 2).

Of note, only one patient showed very high levels of serum NfL (619.86 pg/mL (reference limit ≤ 37.9 pg/mL)) [41]: this was a 73-year-old male with severe hypertrophic cardiomyopathy requiring past ICD implantation. He had NfL dosage assessed about six months after a large-vessel stroke, and in the following months he had also experienced recurrent TIAs.

Mean NfL levels did not differ according to sex (FD males vs. FD females) or clinical presentation (classical vs. late-onset FD patients).

### 3.4. Correlation Analysis Regarding mFS and Serum NfL Levels

The mFS showed a significant direct correlation with AE (*r* = 0.389, *p* = 0.028), NfL levels (*r* = 0.535, *p* = 0.015), creatinine levels (*r* = 0.511, *p* = 0.003), BUN (*r* = 0.367, *p* = 0.039), cystatin C levels (*r* = 0.494, *p* = 0.004), IVS thickness (*r* = 0.504, *p* = 0.003), LV posterior wall thickness (*r* = 0.422, *p* = 0.016), LVMi (*r* = 0.494, *p* = 0.004), RWT (*r* = 0.412, *p* = 0.029), MSSI total score (*r* = 0.493, *p* = 0.004), and general (*r* = 0.354, *p* = 0.047), neurological (*r* = 0.411, *p* = 0.019), cardiac (*r* = 0.386, *p* = 0.029), and renal (*r* = 0.431, *p* = 0.014) MSSI subscores (Supplementary Table S3).

A significant negative correlation was evident with eGFR (*r* = −0.568, *p* < 0.001) (Supplementary Table S3).

The binomial logistic regression model performed to evaluate the effects of age, gender, eGFR, and some known risk factors for cardiovascular disease on the likelihood of having WMLs was statistically significant: χ^2^(1) = 7.697, *p* = 0.006 (Nagelkerke R^2^ 28.9%). Of the variables tested, only eGFR was identified as a statistically significant predictor (OR 0.964, 95% C.I. 0.936 to 0.993, *p* = 0.016): a reduction in eGFR was associated with an increased likelihood of exhibiting an mFS different from 0.

Regarding neuropsychological tests, the mFS showed a significant negative correlation with MMSE score (*r* = −0.410, *p* = 0.022), digit span forward (*r* = −0.373, *p* = 0.039), phonological verbal fluency (*r* = −0.539, *p* = 0.002), and Rey’s complex figure recall (*r* = −0.386, *p* = 0.032).

NfL levels showed a significant positive correlation with AE (*r* = 0.823, *p* < 0.001), DD (*r* = 0.553, *p* = 0.009), plasma lyso-Gb3 levels before the initiation of therapy (*r* = 0.486, *p* = 0.035), creatinine levels (*r* = 0.526, *p* = 0.012), BUN (*r* = 0.493, *p* = 0.020), cystatin C (*r* = 0.674, *p* < 0.001), IVS (*r* = 0.764, *p* < 0.001) and posterior wall thickness (*r* = 0.670, *p* < 0.001), telediastolic (*r* = 0.448, *p* = 0.037) and telesystolic (*r* = 0.491, *p* = 0.020) LV diameter, LVMi (*r* = 0.773, *p* < 0.001), MSSI total score (*r* = 0.816, *p* < 0.001), and cardiac (*r* = 0.804, *p* < 0.001) and renal (*r* = 0.424, *p* = 0.049) MSSI subscores (Supplementary Table S4).

Moreover, NfL values inversely correlated with eGFR values (*r* = −0.772, *p* < 0.001) (Supplementary Table S4).

Regarding cognitive test scores, NfL values significantly correlated inversely with MMSE (*r* = −0.577, *p* = 0.006), digit span forward (*r* = −0.434, *p* = 0.049), Stroop test errors (*r* = −0.697, *p* < 0.001), and MCST category (*r* = −0.460, *p* = 0.041) (Supplementary Table S4).

## 4. Discussion

Our cohort was representative of the gender distribution for FD, confirming a moderate female prevalence (42.5% males vs. 57.5% females), comparable to data from the Fabry Registry (43.5% males vs. 56.5% females) [4,28,42,43].

The core aim of this study was to clinically assess in detail brain involvement and its correlates in FD, in order to understand determining and protective factors that might contribute to increased cerebrovascular risk and the development of cognitive impairment in such patients. To this end, firstly, we assessed prevalence and severity of CSVD by the mFS, and then we correlated these findings with other main disease manifestations, particularly kidney and heart involvement, to verify if specific FD-related comorbidities known to increase the cerebrovascular risk in the general population might further increase cerebrovascular damage in FD patients.

In the general population [44], WMLs are frequently observed. However, the prevalence and severity of WMLs in FD align with those in individuals from the general population at least one to three decades older [44,45], supporting this being actually a characteristic neurological feature of FD.

To estimate global WML burden, we assessed conventional 1.5 T brain MRI images using the modified Fazekas score (mFS), a tool widely used in clinical research.

More than half of our FD patients had some evidence of WMLs, with about 40% showing an mFS ≥1. By comparing our results with previous studies on FD cohorts comparable either in terms of approach to assess WMLs or proportion of treated patients, they are similar to those of Üçeyler et al. [46] and Fellgiebel et al. [47], whereas Korsholm et al. [48] found a higher percentage of patients with significant WMLs (58%). Overall, these data confirm that CSVD is a frequent finding in FD patients.

Regarding the severity of WML load, only a minority of our FD patients (18.8%) showed a moderate-to-severe mFS (≥2). Our findings are close to that found by Korsholm et al. [48], whereas Üçeyler et al. [46] found a lower proportion of FD with mFS ≥ 2 (5%) in a cohort of 87 FD patients. Another study performed on 25 patients found prevalence of mFS ≥ 2 close to 30% [49]. Such discrepancies are possibly due to differences in the sample size or in the mean age of enrolled FD patients.

In our cohort, 4 out of 40 patients (10%) experienced major cerebral events (ischemic stroke), a prevalence similar to that found in other studies with larger patient cohorts [15,50].

Statistical analysis documented, in agreement with the literature [16], that the severity of the mFS was not influenced by gender or disease-modifying therapies (DMTs). Interestingly, a recent meta-analysis on FD supported a beneficial effect of ERT on stroke prevention [51], and our findings suggest that treatment might lower the risk of stroke related to large-vessel occlusion (LVO) rather than lacunar stroke due to CSVD. The potential effect of DMT on WMLs in FD needs to be further investigated, as literature data so far report discordant results [12,15].

Correlation studies between WML load and any features potentially influencing the severity of CSVD showed that WML load increases with age and can even precede the onset of neurological symptoms in FD [52,53]. Accordingly [16], in our FD cohort, mFS showed a positive correlation with the age at evaluation.

Moreover, as shown by Zhao et al. [11], the severity of WML correlated with the overall disease severity: in particular, the positive correlation between mFS and cardiac and renal MSSI subscores supports a critical role of vascular dysfunction in the pathogenesis of brain perfusion changes in FD.

An association between WML burden and LV hypertrophy has been described in the general population [54,55], yet a systematic review focused on determinants of WMLs in FD did not support a similar relationship in this latter disorder [16].

In our FD cohort, WML load positively correlated with LVMi, an echocardiographic parameter assessing LV mass. Also, WMLs correlated with IVS and posterior wall thickness. To the best of our knowledge, the association between mFS and echocardiographic parameters has so far been evaluated only by Esposito et al. [56], who found a correlation between CSVD severity and peak atrial longitudinal strain (PALS), an echocardiographic parameter measuring deformation of the left atrium. These associations between WMLs and echocardiography changes could be explained by the occurrence of a parallel accumulation of Gb3 in both heart and brain tissue as part of multiorgan involvement of FD.

In our study cohort, mFS also correlated with worsening of all renal functionality indices. These findings agree with those of Steinicke et al. [57], who found similar correlations between WML load and eGFR in a large prospective cohort of about 2000 FD patients with early ischemic stroke. On the contrary, Üçeyler et al. [46] did not find a similar correlation in a cohort of 87 FD patients, but the larger proportion of female patients in this cohort could explain this discrepancy. Finally, in agreement with the literature [15,52], we did not find any correlations between FS and common cerebrovascular risk factors.

On logistic regression, eGFR was the only predictor of WMLs. A possible explanation might reside in a shared pathophysiology of microvascular damage [57], likely due to structural and functional similarities regarding small vessels of the human brain and kidney, as already pointed out by some authors [57].

CSVD is known to be associated with cognitive damage [58]. In FD, the prevalence of cognitive impairment ranges from 0 to 30% of patients, mainly affecting executive functioning, processing speed, and attention [19,22,24,59,60].

We assessed the cognitive profile in our FD cohort and its correlations with WML load: almost two-thirds of our FD cohort showed some signs of cognitive impairment, either in attention or executive functions, but also in tests exploring verbal memory and phonological and semantic verbal fluency [19,22].

After correction for age and educational level, we found an inverse correlation between the mFS and test scores exploring global cognitive functioning (MMSE), attention, verbal fluency, and visual episodic memory. In fact, the pattern of neuropsychological impairment and its correlations with the mFS support a predominant cerebrovascular damage rather than neurodegeneration triggered by Gb3 accumulation.

A novel, relevant finding from our study concerns the value of circulating NfL as a biomarker of neuroaxonal damage in FD. In slowly progressive disorders such as FD, the availability of an objective, reproducible biomarker to monitor individual tissue damage with time and eventual response to treatments is relevant for both clinical research and practice purposes. To date, lyso-Gb3 represents the only specific FD biomarker, although with limited sensitivity in female carriers [61]. A few studies [12,47] have applied 3T MRI to assess brain involvement in FD, confirming that DTI is more sensitive in detecting early microstructural white matter changes. However, the use of 3T MRI in FD for clinical research purposes could be hampered by limited individual compliance (e.g., claustrophobia) and also by co-occurrence of FD-related cardiac complications requiring implantation of devices. Therefore, a circulating biomarker may overcome such limitations.

For these reasons, we also assessed the role of circulating NfL as a potential biomarker of CNS damage in FD. Our preliminary results are actually promising, as in fact our FD patient cohort (22 individuals tested) exhibited higher NfL values on average compared to the control group, showing a trend toward significance (*p* = 0.08). Of note, our cohort was relatively young, ruling out the contribution of physiological aging to NfL release [62]. Besides age, occurring also in normal individuals, serum NfL levels directly correlated with pre-therapy lyso-Gb3 levels, disease duration and MSSI total and cardiac and renal subscores. The lack of correlation of NfL levels with the general and neurological MSSI subscores is not surprising, as these scores reflect the burden from other disease features (e.g., gastrointestinal symptoms, diaphoresis, small fiber-type neuropathy) not expected to affect NfL levels [63]. Also, NfL levels correlated with alterations of all echocardiographic parameters and with worsening of renal functioning. These results are in agreement with the hypothesis stated earlier, namely, that CNS damage progression would be concomitant with general progression [11].

Regarding cognitive impairment, NfL levels inversely correlated with deterioration of specific cognitive performances, as in various other neurological disorders [64,65,66]. Similar correlations of NfL levels with cognitive abilities and lyso-Gb3 levels were documented in the only study so far performed that analyzed NfL levels in a small cohort of 12 female FD patients [67]. Differently from our results, that study found no differences in NfL levels between FD patients and controls, though using a low-resolution enzyme-linked immunosorbent assay (ELISA). Further studies on larger and well-characterized cohorts are needed to definitively establish the value of NfL as a biomarker of brain damage in FD.

The main advantage of this study is represented by the reduction in the risk of bias in the results related to technical or laboratory differences: in fact, all our patients have been homogeneously assessed in one tertiary center and by the same specialty clinicians (i.e., cardiologists, neurologists, nephrologists etc.). Also, we used the same laboratory facilities to assess circulating biomarkers included in the study.

Regarding NfL determination, we used high-resolution SiMoA, which represents the gold standard for its diagnostic assessment.

The main limitation is in the assessment of WMLs by the mFS, a score that is both subjective and operator-dependent. To reduce this risk of bias, the mFS was assessed blinded by two trained neurologists, and actually did not show discrepancies in their scoring.

The small sample of our study cohort, particularly regarding NfL assessment, represents another limitation, which does not allow us to draw definite conclusions about its potential role as a biomarker of CNS involvement in FD.

## 5. Conclusions

In conclusion, our study confirms that mild-to-moderate CSVD is a characteristic brain “signature” in FD patients, regardless of gender and phenotype, but coherent with overall disease severity. In particular, both cardiac and renal involvement correlate with WML load, but only renal involvement appears to be predictive of CNS damage in FD, likely due to anatomical and functional similarities between their respective vascularization, rather than depending on the effect on kidney function deterioration on cerebrovascular risk factors, such as systemic arterial hypertension. Brain microvascular damage is also associated with mild cognitive impairment in FD. Of note, NfL might represent a potential sensitive and easy-to-perform biomarker of CNS damage in FD, either to monitor disease progression and response to treatments, but additional studies on larger samples are needed to clarify this issue.

## Figures and Tables

**Table 1:** Demographic and disease-related features of the cohort. All the frequencies were calculated on the whole sample (*n* = 40) unless otherwise specified. Abbreviations: AAD, age at diagnosis; AAO, age at onset; AE, age at evaluation; BUN, blood urea nitrogen; DD, disease duration; eGFR, estimated glomerular filtration rate; ERT, enzymatic replacement therapy; IQR, interquartile range; IVS, interventricular septum; LV, left ventricular; LVMi, left ventricular mass index; MSSI, Mainz Severity Score Index; NfL, neurofilament light chain; PNP, polyneuropathy; RWT, relative wall thickness; sAH, systemic arterial hypertension; SD, standard deviation; VAS, visual analogue scale; y, years.

	*n* (%)	Mean	SD	Median	IQR
**Males**	17 (42.5%)				
**AE, y**		45.38	16.93	44.17	25.00
**AAO, y**		26.90	17.53	24.00	25.00
**AAD, y**		35.35	17.83	35.50	27.00
**DD, y**		19.41	17.30	12.50	25.80
**Diagnostic delay, y**		10.53	12.91	5.00	18.00
**Phenotype**	classic 24 (60.00%)				
	late-onset 16 (40%)				
***GLA* variant**	missense 31 (77.50%)				
	truncating 9 (22.50%)				
**Treatment (*n* = 32)**	ERT 25 (78.10%)				
	chaperone 6 (18.80%)				
**Treatment duration, y (*n* = 32)**		8.50	5.53	8.00	5.00
**MSSI**		17.80	14.30	14.50	14.00
** general**		4.08	3.17	3.00	4.00
** renal**		3.55	5.44	1.00	4.00
** cardiological**		5.48	5.79	3.00	9.00
** neurological**		4.38	3.49	3.50	6.00
**Lyso-Gb3 (before treatment, ng/mL (*n* = 34)**		11.27	14.58	5.35	8.38
**Lyso-Gb3 over the upper limit (before treatment) *n* = 34**	31 (91.20%)				
**Lyso-Gb3 (after treatment, ng/mL (*n* = 34)**		8.90	12.08	4.10	6.90
**Lyso-Gb3 over the upper Limit (after treatment) *n* = 34**	27 (79.40%)				
**Alpha-Gal A activity, % (*n* = 39)**		28.86	21.68	20.00	41.56
**Renal features**					
**Renal dysfunction**	20 (50.00%)				
**Creatinine, mg/dL**		1.01	0.47	0.83	0.53
**Cystatin C, mg/dL**		0.99	0.52	0.86	0.37
**BUN, mg/dL**		19.45	10.91	15.50	12.00
**eGFR, ml/min**		89.12	33.83	90.40	54.60
**24 h proteinuria, mg (*n* = 26)**		63.48	126.33	0.12	74.05
**24 h albuminuria, mg (*n* = 37)**		130.64	290.63	27.00	83.50
**Microalbuminuria (*n* = 36)**	17 (47.20%)				
**Major renal events**	4 (10.00%)				
**Cardiovascular features**					
**Loop-recorder implantation**	3 (7.50%)				
**PMK/ICD**	8 (20.00%)				
**Major cardiovascular events**	9 (22.50%)				
**sAH**	22 (55.00%)				
**Dyslipidemia**	12 (30.00%)				
**Statin treatment**	11 (27.50%)				
**Antithrombotic drug treatment**	9 (22.50%)				
**Smoking habit**	16 (40.00%)				
**Diabetes mellitus**	2 (5.00%)				
**Lower limb edema**	4 (10.00%)				
**IVS thickness, mm**		13.29	5.57	12.00	6.00
**LV end-diastolic diameter, mm**		45.50	5.20	46.00	7.90
**LV end-systolic diameter, mm**		27.06	4.60	26.70	5.90
**LV posterior wall thickness, mm**		11.52	3.55	10.50	5.00
**LVMi g/m^2^**		123.50	60.86	102.50	62.00
**LV hypertrophy**	18 (45.00%)				
**RWT (*n* = 35)**		0.45	0.16	0.41	0.31
**Neurological features**					
**Previous stroke**	4 (10%)				
	2 (50.00%) lacunar stroke				
	2 (50.00%) large-vessel stroke				
**Brain MRI pulvinar hyperintensity (*n* = 32)**	1 (3.13%)				
**Brain MRI basilar dolichoectasia (*n* = 32)**	3 (2.88%)				
**Fazekas score (*n* = 32)**					
**0**	19 (59.38%)				
**1**	7 (21.88%)				
**2**	3 (9.38%)				
**3**	3 (9.38%)				
**Serum NfL (pg/mL) (*n* = 22)**		45.59	129.36	11.50	17.65
**PNP**	1 (2.5%)				
**Depression**	12 (30.00%)				
**Antidepressive treatment**	3 (7.50%)				
**Headache**	15 (37.50%)				
**VAS score**		1.68	1.70	1.00	3.00
**Tinnitus**	11 (27.50%)				
**Vertigo**	9 (22.50%)				
**Hearing loss**	13 (32.50%)				
**Acroparesthesias**	29 (72.50%)				
**Dyshidrosis**	23 (57.50%)				
**Other**					
**Recurrent fever**	13 (32.50%)				
**Angiokeratomas**	13 (32.50%)				
**Corneal abnormalities**	12 (30.00%)				
**Gastrointestinal manifestations**	21 (52.50%)				

**Table 2:** Descriptive statistics of the cognitive test scores (raw and adjusted for years of education and age) in the study cohort (*n* = 39). Abbreviations: adj, adjusted; max., maximum; MMSE, Mini-Mental State Examination; MCST, modified card sorting test; MFTC, multiple feature target cancellation; min., minimum; RAVLT, Rey auditory verbal learning test; SD, standard deviation.

	Mean	SD	Median	Min.	Max.
**MMSE**	28.79	1.64	29.00	22.00	30.00
**RAVLT immediate recall (adj)**	40.67 (36.77)	12.75 (8.90)	42.00 (35.02)	10.00 (19.14)	65.00 (57.80)
**RAVLT delayed recall (adj)**	8.87 (7.74)	3.17 (2.17)	10.00 (7.81)	0 (0)	14.00 (11.80)
**RAVLT forced-choice recognition (adj)**	0.91	0.11	0.95	0.53	1.00
**Rey’s complex figure recall (adj)**	17.53 (13.95)	7.03 (6.07)	17.50 (15.71)	2.50 (0.34)	29.00 (25.40)
**Digit span forward (adj)**	5.97 (5.74)	1.20 (1.11)	6.00 (5.63)	4 (3.66)	8 (7.73)
**Digit span backward (adj)**	4.08 (3.77)	1.04 (0.96)	4.00 (3.62)	2 (1.53)	6 (5.81)
**Spatial span forward (adj)**	5.00 (4.74)	0.89 (0.82)	5.00 (4.68)	4 (3.35)	7 (6.46)
**Spatial span backward (adj)**	4.46 (4.21)	0.79 (0.73)	4.00 (3.94)	3 (3.26)	7 (6.29)
**Raven’s colored progressive matrices (adj)**	29.69 (27.35)	30.00 (27.31)	4.92 (3.89)	16 (18.37)	35 (34.35)
**Rey’s complex figure copy (adj)**	31.33 (30.43)	4.67 (3.73)	33.00 (31.40)	18.00 (19.22)	36.00 (37.24)
**MFTC accuracy adj.**	0.91	0.15	0.96	0.09	1.00
**MFTC false (adj)**	0.10 (0.53)	0.31 (0.48)	0.00 (0.42)	0 (0)	1 (2.85)
**MFTC time (adj)**	40.45 (40.43)	12.27 (18.20)	37.00 (40.78)	24.00 (0.82)	72.00 (77.34)
**Phonological verbal fluency (adj)**	36.36 (32.39)	14.32 (13.17)	38.00 (31.38)	11.00 (3.70)	71.00 (71.07)
**Categorial verbal fluency—total (adj)**	18.72 (14.58)	5.82 (5.12)	20.00 (14.13)	7.00 (4.77)	28.00 (23.59)
**Stroop test time (adj)**	19.96 (23.55)	11.50 (10.32)	18.00 (21.74)	6.50 (3.29)	58.00 (60.57)
**Stroop test errors (adj)**	0.15 (0.94)	0.49 (0.93)	0 (0.81)	0 (0)	2 (3.25)
**MCST—category**	5.47	1.18	6.00	1	6
**MCST—perseverative errors (adj)**	2.29 (2.56)	3.72 (3.43)	1.00 (1.29)	0 (0)	16.00 (15.19)

## Data Availability

The data presented in this study are available on reasonable request from the corresponding author.

## Supplementary Materials

The following supporting information can be downloaded at https://www.mdpi.com/article/10.3390/brainsci15020166/s1. Table S1: *GLA* variants found in our cohort; Table S2: Comparison between male and female FD patients in our study cohort (*n* = 40); Table S3: Spearman correlation coefficients between modified Fazekas score and the main relevant demographic and clinical parameters; Table S4: Spearman correlation coefficients between NfL levels and the main relevant demographic and clinical parameters.
