# Supplementary material for: Prevalence and Clinical Correlates of Cerebrovascular Alterations in Fabry Disease: A Cross-Sectional Study
Source: Brain Sci. 2025 Feb 7;15(2):166. doi: 10.3390/brainsci15020166 (PMC11852458; doi:10.3390/brainsci15020166)
Supplement: Supplementary file 1 [file brainsci-15-00166-s001.zip › brainsci-3430399-supplementary/Supplementary table S1.pdf]

Table S1. *GLA* variants found in our cohort.

Patient code	GLA variant (NM_000169.2)	Amino acid substitution
RM1A	c.758T>C	p.(Ile253Thr)
RM2W	c.801+1G>T; IVS5+1G>T	
RM5F	c.668G>A	p.(Cys233Tyr)
RM6F	c.644A>G	p.(Ans215Ser)
RM7K	c.718_719del	p.(Lys240Glufs)
RM10N	c.644A>G	p.(Ans215Ser)
RM11B	c.548G>C	p.(Gly183Ala)
RM12T	c.548G>C	p.(Gly183Ala)
RM13Y	c.548G>C	p.(Gly183Ala)
RM14L	c.801+1G>T; IVS5+1G>T	
RM15S	c.126_127insCA	p.(Gly43Hisfs*14)
RM16G	c.126_127insCA	p.(Gly43Hisfs*14)
RM17H	c.901C>G	p.(Arg301Gly)
RM20D	c.337T>C	p.(Phe113Leu)
RM21S	c.758T>C	p.(Ile253Thr)
RM22A	c.758T>C	p.(Ile253Thr)
RM23U	c.644A>G	p.(Ans215Ser)
RM25J	c907A>T	p.(Ile303Phe)
RM27P	c.6444A>G	p.(Asn215Ser)
RM29N	c.644 A>G	p.(Asn215Ser)
RM30A	c.1094dupA	p.(Tyr365*)
RM32Q	c.644A>G	p.(Ans215Ser)
RM33R	c.1284_1287delACTT	p.(Leu428Phefs)
RM34K	c.548G>C	p.(Gly183Ala)
RM37L	c.644A>G	p.(Ans215Ser)
RM38L	c.644A>G	p.(Ans215Ser)
RM40Q	c.644A>G	p.(Ans215Ser)

RM41G	c.644A>G	p.(Ans215Ser)
RM44L	c.747C>A	p.(Ans249Lys)
RM45K	c.747C>A	p.(Ans249Lys)
RM46A	c.747C>A	p.(Ans249Lys)
RM47G	c.747C>A	p.(Ans249Lys)
RM48Y	c.747C>A	p.(Ans249Lys)
RM54A	c.547+1G>T	
RM55R	c.547+1G>T	
RM56Y	c.644A>G	p.(Ans215Ser)
RM59R	c.337T>C	p.(Phe113Leu)
RM61Q	c.668G>A	p.(Cys233Tyr)
RM63R	c.668G>A	p.(Cys233Tyr)
RM65H	c.6444A>G	p.(Asn215Ser)
