# Supplementary material for: Prevalence and Clinical Correlates of Cerebrovascular Alterations in Fabry Disease: A Cross-Sectional Study
Source: Brain Sci. 2025 Feb 7;15(2):166. doi: 10.3390/brainsci15020166 (PMC11852458; doi:10.3390/brainsci15020166)
Supplement: Supplementary file 1 [file brainsci-15-00166-s001.zip › brainsci-3430399-supplementary/Supplementary table S2.pdf]

Table S2. Comparison between male and female FD patients regarding the different variables found in our study cohort (n=40). Abbreviations: AAD, Age at diagnosis; AAO, age at onset; AE, age at evaluation; BUN, blood urea nitrogen; DD, disease duration; eGFR, estimated Glomerular Filtration Rate; ERT, enzymatic replacement therapy; IQR, interquartile range; IVS, interventricular septum; LV, left ventricular; LVMI, left ventricular mass index; MSSI, Mainz Severity Score Index; ND, not done; NfL, Neurofilament light chain; PNP, polyneuropathy; RWT, relative wall thickness; sAH, systemic arterial hypertension; SD, standard deviation; VAS, Visual Analog Scale; y, years.

	**Males (n=17)**		**Females (n=23)**		**p**
	**n (%)**	**mean±SD, median (IQR)**	**n (%)**	**mean±SD, median (IQR)**	
**AE, y**		(n=17) 50.70±15.56, 55.58 (21.63)		(n=23) 44.21±18.91, 42.83 (24.42)	0.191
**AAO, y**		(n=17) 27.29±18.31, 26.00 (35.00)		(n=23) 26.61±17.34, 24.00 (25.00)	0.989
**AAD, y**		(n=17) 40.06±17.75, 41.00 (28.00)		(n=23) 35.35±18.02, 31.00 (28.00)	0.342
**DD, y**		(n=17) 22.71±18.34, 15.00 (32.50)		(n=23) 16.98±16.48, 11.00 (19.00)	0.265
**Diagnostic delay, y**		(n=17) 11.71±13.70, 6.00 (17.00)		(n=23) 9.65±12.54, 2.00 (18.00)	0.401
**GLA variant (missense)**<br>**GLA variant (truncating)**	11/17 (64.70%)<br>6/17 (35.3%)		20/23 (87.0%)<br>3/23 (13.0%)		0.134
**Phenotype (classic)**	9/17 (52.90%)		15/23 (65.20%)		0.522
**Phenotype (late-onset)**	8/17 (47.10%)		8/23 (34.80%)		0.522
**Treatment (ERT or chaperone)**	15/17 (88.20%)		17/23 (73.90%)		0.428
**Duration of treatment, y**		(n=15) 9.60±6.95, 8.00 (13.00)		(n=17) 7.53±3.86, 8.00 (5.00)	0.602
**MSSI**<br>**general**<br>**renal**<br>**cardiological**<br>**neurological**		(n=17) 26.35±17.16, 21.00 (29.00)<br>5.29±3.89, 4.00 (6.00)<br>6.24±7.10, 4.00 (12.00)<br>9.71±5.63, 10.00 (11.00)<br>5.12±3.98, 5.00 (6.00)		(n=23) 11.48±7.07, 10.00 (11.00)<br>3.17±2.19, 3.00 (4.00)<br>1.57±2.47, 0 (3.00)<br>2.35±4.50, 1.00 (3.00)<br>3.83±3.06, 3.00 (5.00)	**0.003**<br>0.090<br>**0.025**<br>**<0.001**<br>0.342
**lyso-Gb3 (before treatment), ng/ml**		(n=16) 19.83±17.74, 12.30 (28.83)		(n=18) 3.67±2.31, 3.15 (3.65)	**<0.001**

**lyso-Gb3 (after treatment), ng/ml**		(n=14) 16.91±15.66, 10.55 (27.20)		(n=20) 3.29±2.28, 2.75 (3.20)	**0.001**
**α-Gal A activity, %**		(n=17) 9.56±5.59, 7.66 (9.37)		(n=22) 43.77±17.11, 48.40 (27.22)	**<0.001**
			**Renal features**		
**Renal dysfunction**	13/17 (76.50%)		7/23 (30.40%)		**0.004**
**Creatinine, mg/dl**		(n=17) 1.21±0.41, 1.15 (0.51)		(n=23) 0.85±0.46, 0.71 (0.23)	**0.001**
**Cystatine C, mg/dl**		(n=17) 1.11±0.47, 1.01 (0.59)		(n=23) 0.90±0.55, 0.80 (0.21)	**0.015**
**BUN, mg/dl**		(n=17) 21.88±10.42, 21.00 (15.00)		(n=23) 17.65±11.15, 14.00 (7.00)	0.085
**eGFR, ml/min**		(n=17) 76.55±32.14, 66.90 (43.90)		(n=23) 98.40±32.64, 108.80 (37.70)	**0.030**
**24-h proteinuria, mg**		(n=12) 59.26±110.11, 16.50 (61.50)		(n=14) 67.09±142.83, 0.01 (112.90)	0.705
**24-h albuminuria, mg**		(n=16) 160.31±206.69, 46.50 (319.75)		(n=21) 108.03±344.61, 11.00 (36.50)	0.130
**microalbuminuria**	11/15 (73.30%)		6/21 (28.60%)		**0.008**
**Major renal events**	3/17 (17.60%)		1/23 (4.30%)		0.294
			**Cardiac features**		
**PMK/ICD implantation**	7/17 (41.20%)		1/23 (4.30%)		**0.006**
**Loop-recorder implantation**	0/17 (0%)		3/23 (13.00%)		0.248
**Major cardio-vascular events**	8/17 (47.10%)		1/23 (4.30%)		**0.002**
**sAH**	13/17 (76.50%)		9/23 (39.10%)		**0.019**
**Dyslipidemia**	6/17 (35.30%)		6/23 (26.10%)		0.530
**Statin treatment**	7/17 (41.20%)		4/23 (17.40%)		0.153
**Antithrombotic drug treatment**	6/17 (35.30%)		3/23 (13.00%)		0.134
**Smoking habit**	6/17 (35.30%)		10/23 (43.50%)		0.601
**Diabetes mellitus**	2/17 (0.174%)		0/23 (0%)		0.174
**Lower limb oedema**	2/17 (11.80%)		2/23 (8.70%)		1
**IVS thickness, mm**		(n=17) 17.09±6.38, 15.00 (11.30)		(n=23) 10.48±2.47, 9.00 (3.40)	**<0.001**

**LV telediastolic diameter, mm**		(n=17) 47.16±4.48, 47.00 (5.50)		(n=23) 44.27±5.44, 45.00 (7.30)	0.095
**LV telesistolic diameter, mm**		(n=17) 29.13±5.01, 28.30 (7.50)		(n=23) 25.53±3.68, 24.00 (5.10)	**0.016**
**LV posterior wall thickness, mm**		(n=17) 13.81±3.66, 14.00 (5.60)		(n=23) 9.84±2.38, 9.00 (4.00)	**<0.001**
**LVMI, g/m^2**		(n=17) 166.24±69.51, 145.00 (122)		(n=23) 91.91±24.47, 86.00 (26)	**<0.001**
**LV hypertrophy**	12/17 (70.60%)		6/23 (26.10%)		**0.005**
**RWT**		(n=12) 0.57±0.13, 0.60 (0.14)		(n=23) 0.38±0.13, 0.32 (0.23)	**<0.001**
**Neurological features**					
**Previous stroke**	2/17 (11.80%)		2/23 (8.70%)		1
**Brain MRI pulvinar hyperintensity**	1/15 (6.70%)		0/17 (0%)		0.469
**Brain MRI basilar dolichoectasia**	3/15 (20%)		0/17 (0%)		0.092
**Fazekas score**		(n=15) 0.93±1.16, 0 (2.00)		(n=17) 0.47±0.80, 0 (1.00)	0.370
**NfL, pg/ml**		(n=11) 78.33±180.78, 21.97 (38.16)		(n=11) 12.86±10.24, 10.46 (5.80)	0.116
**PNP**	/ (%)		/ (%)		
**Depression**	5/17 (29.40%)		7/23 (30.40%)		0.944
**Anti-depressive treatment**	1/17 (5.90%)		2/23 (8.70%)		1
**Headache**	7/17 (41.20%)		8/23 (34.80%)		0.680
**VAS**		(n=17) 1.76±1.95, 1.00 (4.00)		(n=23) 1.61±1.53, 1.00 (3.00)	0.957
**Tinnitus**	5/17 (29.40%)		6/23 (26.10%)		1
**Vertigo**	4/17 (23.50%)		5/23 (21.70%)		1
**Hearing loss**	8/17 (47.10%)		5/23 (21.70%)		0.091
**Other**					
**Acroparesthesias**	11/17 (64.70%)		18/23 (78.30%)		0.477
**Dysidrosis**	9/17 (52.90%)		14/23 (60.90%)		0.616
**Recurrent fever**	10/17 (58.80%)		3/23 (13.00%)		**0.002**
**Angiokeratomas**	7/17 (41.20%)		6/23 (26.10%)		0.314
**Corneal abnormalities**	4/17 (23.50%)		8/23 (34.80%)		0.443

**Gastro-intestinal manifestations**	7/17 (41.20%)		14/23 (60.90%)		0.218
