# Supplementary material for: Prevalence and Clinical Correlates of Cerebrovascular Alterations in Fabry Disease: A Cross-Sectional Study
Source: Brain Sci. 2025 Feb 7;15(2):166. doi: 10.3390/brainsci15020166 (PMC11852458; doi:10.3390/brainsci15020166)
Supplement: Supplementary file 1 [file brainsci-15-00166-s001.zip › brainsci-3430399-supplementary/Supplementary table S3.pdf]

Table S3. Spearman correlation coefficient between modified Fazekas Score and the main relevant demographic and clinical parameters. Abbreviations: AAD, Age at diagnosis; AAO, age at onset; AE, age at evaluation; BUN, blood urea nitrogen; DD, disease duration; eGFR, estimated Glomerular Filtration Rate; LV, left ventricular; LVMI, left ventricular mass index; MMSE, Mini Mental State Examination; MCST, Modified Card Sorting Test; MFTC, Multiple Features Targets Cancellation; RAVLT, Rey Auditory Verbal Learning test; MSSI, Mainz Severity Score Index; NfL, neurofilament light chain; RWT, relative wall thickness; VAS, Visual Analog Scale; y, years.

		Fazekas Score
Fazekas Score	Correlation coefficient	
	Sig. (two-tailed)	
	N	
AE	Correlation coefficient	.389*
	Sig. (two-tailed)	.028
	N	32
Alpha GAL A (activity %)	Correlation coefficient	-.307
	Sig. (two-tailed)	.087
	N	32
Serum NfL	Correlation coefficient	.535*
	Sig. (two-tailed)	.015
	N	20
AAO	Correlation coefficient	.198
	Sig. (two-tailed)	.278
	N	32
DD	Correlation coefficient	.166
	Sig. (two-tailed)	.364
	N	32
LysoGb3 before therapy (ng/ml)	Correlation coefficient	.282
	Sig. (two-tailed)	.131
	N	30
LysoGb3 after therapy (ng/ml)	Correlation coefficient	.184
	Sig. (two-tailed)	.348
	N	28
Years of treatment	Correlation coefficient	.203
	Sig. (two-tailed)	.330
	N	25
Creatinine (mg/dl)	Correlation coefficient	.511**
	Sig. (two-tailed)	.003

	N	32
BUN	Correlation coefficient	.367*
	Sig. (two-tailed)	.039
	N	32
Cystatin C	Correlation coefficient	.494**
	Sig. (two-tailed)	.004
	N	32
eGFR	Correlation coefficient	-.568**
	Sig. (two-tailed)	<.001
	N	32
Proteinuria 24h (mg/dl)	Correlation coefficient	-.199
	Sig. (two-tailed)	.386
	N	21
Albuminuria 24h (mg)	Correlation coefficient	.024
	Sig. (two-tailed)	.902
	N	29
LV septum (mm)	Correlation coefficient	.504**
	Sig. (two-tailed)	.003
	N	32
LV telediastolic diameter	Correlation coefficient	.143
	Sig. (two-tailed)	.434
	N	32
LV telesistolic diameter	Correlation coefficient	.036
	Sig. (two-tailed)	.846
	N	32
Posterior wall thickness	Correlation coefficient	.422*
	Sig. (two-tailed)	.016
	N	32
LVMI (g/m2)	Correlation coefficient	.494**
	Sig. (two-tailed)	.004
	N	32
RWT	Correlation coefficient	.421*
	Sig. (two-tailed)	.029
	N	27
MSSI	Correlation coefficient	.493**
	Sig. (two-tailed)	.004
	N	32
General (MSSI)	Correlation coefficient	.354*

	Sig. (two-tailed)	.047
	N	32
Neurological (MSSI)	Correlation coefficient	.411*
	Sig. (two-tailed)	.019
	N	32
Cardiac (MSSI)	Correlation coefficient	.386*
	Sig. (two-tailed)	.029
	N	32
Renal (MSSI)	Correlation coefficient	.431*
	Sig. (two-tailed)	.014
	N	32
VAS	Correlation coefficient	.081
	Sig. (two-tailed)	.661
	N	32
Years of education	Correlation coefficient	.059
	Sig. (two-tailed)	.754
	N	31
MMSE	Correlation coefficient	-.410*
	Sig. (two-tailed)	.022
	N	31
RAVLT immediate recall (adj)	Correlation coefficient	-.311
	Sig. (two-tailed)	.089
	N	31
RAVLT delayed recall (adj)	Correlation coefficient	-.242
	Sig. (two-tailed)	.190
	N	31
RAVLT forced choice recognition (adj)	Correlation coefficient	-.204
	Sig. (two-tailed)	.270
	N	31
Digit span forward (adj)	Correlation coefficient	-.373*
	Sig. (two-tailed)	.039
	N	31
Digit span backward (adj)	Correlation coefficient	.011
	Sig. (two-tailed)	.953
	N	31
Spatial span forward (adj)	Correlation coefficient	-.100
	Sig. (two-tailed)	.592
	N	31

Spatial span backward (adj)	Correlation coefficient	-.054
	Sig. (two-tailed)	.774
	N	31
Raven’s coloured progressive matrices (adj)	Correlation coefficient	-.153
	Sig. (two-tailed)	.411
	N	31
MFTC false (adj)	Correlation coefficient	-.193
	Sig. (two-tailed)	.299
	N	31
MFTC time (adj)	Correlation coefficient	.086
	Sig. (two-tailed)	.646
	N	31
MFTC accuracy	Correlation coefficient	-.012
	Sig. (two-tailed)	.950
	N	31
Phonological verbal fluency (adj)	Correlation coefficient	-.539**
	Sig. (two-tailed)	.002
	N	31
Categorial verbal fluency (adj)	Correlation coefficient	-.249
	Sig. (two-tailed)	.177
	N	31
Stroop test time (adj)	Correlation coefficient	-.069
	Sig. (two-tailed)	.710
	N	31
Stroop test errors (adj)	Correlation coefficient	-.208
	Sig. (two-tailed)	.261
	N	31
Rey’s complex figure copy (adj)	Correlation coefficient	-.242
	Sig. (two-tailed)	.189
	N	31
Rey’s complex figure recall (adj)	Correlation coefficient	-.386*
	Sig. (two-tailed)	.032
	N	31
MCST – Category	Correlation coefficient	.032
	Sig. (two-tailed)	.868
	N	30
MCST – Perseverative errors (adj)	Correlation coefficient	-.234
	Sig. (two-tailed)	.213

N	30
