# Supplementary material for: Prevalence and Clinical Correlates of Cerebrovascular Alterations in Fabry Disease: A Cross-Sectional Study
Source: Brain Sci. 2025 Feb 7;15(2):166. doi: 10.3390/brainsci15020166 (PMC11852458; doi:10.3390/brainsci15020166)
Supplement: Supplementary file 1 [file brainsci-15-00166-s001.zip › brainsci-3430399-supplementary/Supplementary table S4.pdf]

Table S4. Spearman correlation coefficient between NfL levels and the main relevant demographic and clinical parameters. Abbreviations: AAD, Age at diagnosis; AAO, age at onset; AE, age at evaluation; BUN, blood urea nitrogen; DD, disease duration; eGFR, estimated Glomerular Filtration Rate; LV, left ventricular; LVMI, left ventricular mass index; MMSE, Mini Mental State Examination; MCST, Modified Card Sorting Test; MFTC, Multiple Features Targets Cancellation; RAVLT, Rey Auditory Verbal Learning test; MSSI, Mainz Severity Score Index; NfL, neurofilament light chain; RWT, relative wall thickness; VAS, Visual Analog Scale; y, years.

		NfL (pg/ml)
AE	Correlation coefficient	.823**
	Sig. (two-tailed)	<.001
	N	22
Alpha GAL A (activity %)	Correlation coefficient	-.316
	Sig. (two-tailed)	.152
	N	22
AAO	Correlation coefficient	.343
	Sig. (two-tailed)	.118
	N	22
DD	Correlation coefficient	.553**
	Sig. (two-tailed)	.008
	N	22
LysoGb3 before therapy (ng/ml)	Correlation coefficient	.486*
	Sig. (two-tailed)	.035
	N	19
LysoGb3 after therapy (ng/ml)	Correlation coefficient	.373
	Sig. (two-tailed)	.141
	N	17
Years of treatment	Correlation coefficient	.340
	Sig. (two-tailed)	.182
	N	17
Creatinine (mg/dl)	Correlation coefficient	.526*
	Sig. (two-tailed)	.012
	N	22
BUN (mg/dl)	Correlation coefficient	.493*
	Sig. (two-tailed)	.020
	N	22
Cystatin C	Correlation coefficient	.674**
	Sig. (two-tailed)	<.001

	N	22
eGFR (ml/min)	Correlation coefficient	-.772**
	Sig. (two-tailed)	<.001
	N	22
Proteinuria 24h (mg/dl)	Correlation coefficient	-.392
	Sig. (two-tailed)	.166
	N	14
Albuminuria 24h (mg)	Correlation coefficient	.472*
	Sig. (two-tailed)	.031
	N	21
LV septum (mm)	Correlation coefficient	.764**
	Sig. (two-tailed)	<.001
	N	22
LV telediastolic diameter	Correlation coefficient	.448*
	Sig. (two-tailed)	.037
	N	22
LV telesistolic diameter	Correlation coefficient	.491*
	Sig. (two-tailed)	.020
	N	22
Posterior wall thickness	Correlation coefficient	.670**
	Sig. (two-tailed)	<.001
	N	22
LVMI (g/m2)	Correlation coefficient	.773**
	Sig. (two-tailed)	<.001
	N	22
MSSI	Correlation coefficient	.816**
	Sig. (two-tailed)	<.001
	N	22
General (MSSI)	Correlation coefficient	.360
	Sig. (two-tailed)	.100
	N	22
Neurological (MSSI)	Correlation coefficient	.271
	Sig. (two-tailed)	.223
	N	22
Cardiac (MSSI)	Correlation coefficient	.804**
	Sig. (two-tailed)	<.001
	N	22
Renal (MSSI)	Correlation coefficient	.424*

	Sig. (two-tailed)	.049
	N	22
VAS	Correlation coefficient	.078
	Sig. (two-tailed)	.730
	N	22
MMSE	Correlation coefficient	-.577**
	Sig. (two-tailed)	.006
	N	21
RAVLT immediate recall (adj)	Correlation coefficient	-.331
	Sig. (two-tailed)	.143
	N	21
RAVLT delayed recall (adj)	Correlation coefficient	-.196
	Sig. (two-tailed)	.394
	N	21
RAVLT forced choice recognition (adj)	Correlation coefficient	-.334
	Sig. (two-tailed)	.139
	N	21
Digit span forward (adj)	Correlation coefficient	-.434*
	Sig. (two-tailed)	.049
	N	21
Digit span backward (adj)	Correlation coefficient	.019
	Sig. (two-tailed)	.935
	N	21
Spatial span forward (adj)	Correlation coefficient	.105
	Sig. (two-tailed)	.652
	N	21
Spatial span backward (adj)	Correlation coefficient	.241
	Sig. (two-tailed)	.292
	N	21
Raven’s coloured progressive matrices (adj)	Correlation coefficient	-.216
	Sig. (two-tailed)	.346
	N	21
MFTC false (adj)	Correlation coefficient	-.094
	Sig. (two-tailed)	.684
	N	21
MFTC time (adj)	Correlation coefficient	-.262
	Sig. (two-tailed)	.251
	N	21

MFTC accuracy	Correlation coefficient	-.009
	Sig. (two-tailed)	.968
	N	21
Phonological verbal fluency (adj)	Correlation coefficient	-.344
	Sig. (two-tailed)	.127
	N	21
Categorial verbal fluency (adj)	Correlation coefficient	.058
	Sig. (two-tailed)	.801
	N	21
Stroop test time (adj)	Correlation coefficient	-.177
	Sig. (two-tailed)	.444
	N	21
Stroop test errors (adj)	Correlation coefficient	-.697**
	Sig. (two-tailed)	<.001
	N	21
Rey’s complex figure copy (adj)	Correlation coefficient	-.374
	Sig. (two-tailed)	.095
	N	21
Rey’s complex figure recall (adj)	Correlation coefficient	-.153
	Sig. (two-tailed)	.507
	N	21
MCST – Category	Correlation coefficient	-.460*
	Sig. (two-tailed)	.041
	N	20
MCST – Perseverative errors (adj)	Correlation coefficient	.022
	Sig. (two-tailed)	.927
	N	20
Fazekas Score	Correlation coefficient	.535*
	Sig. (two-tailed)	.015
	N	20
Years of education	Correlation coefficient	-.090
	Sig. (two-tailed)	.699
	N	21
